# Supplementary material for: Characterizing the Cellular Response to Nitrogen-Doped Carbon Nanocups
Source: Nanomaterials (Basel). 2019 Jun 16;9(6):887. doi: 10.3390/nano9060887 (PMC6631063; doi:10.3390/nano9060887)
Supplement: Supplementary file 1 [file nanomaterials-09-00887-s001.pdf]

nanomaterials

MDPI

Supplemental Materials

Characterizing the Cellular Response to Nitrogen-Doped Carbon Nanocups

Amber S. Griffith [1], Thomas D. Zhang [1], Seth C. Burkert [2], Zelal Adiguzel [3], Ceyda Acilan [4], Alexander Star [2] and William S. Saunders [1,*]

[1] Department of Biological Sciences, University of Pittsburgh, Pittsburgh, PA 15260, USA; ASG56@pitt.edu (A.S.G.); TDZ7@pitt.edu (T.D.Z.)
[2] Department of Chemistry, University of Pittsburgh, Pittsburgh, PA 15260, USA; scb55@pitt.edu (S.C.B.); astar@pitt.edu (A.S.)
[3] TUBITAK, Marmara Research Center, Genetic Engineering and Biotechnology Institute, Gebze/Kocaeli 41470, Turkey; zelal.adiguzel@tubitak.gov.tr
[4] School of Medicine, Koc University, 34450 Sarıyer, Turkey; cayhan@ku.edu.tr
* Correspondence: wsaund@pitt.edu

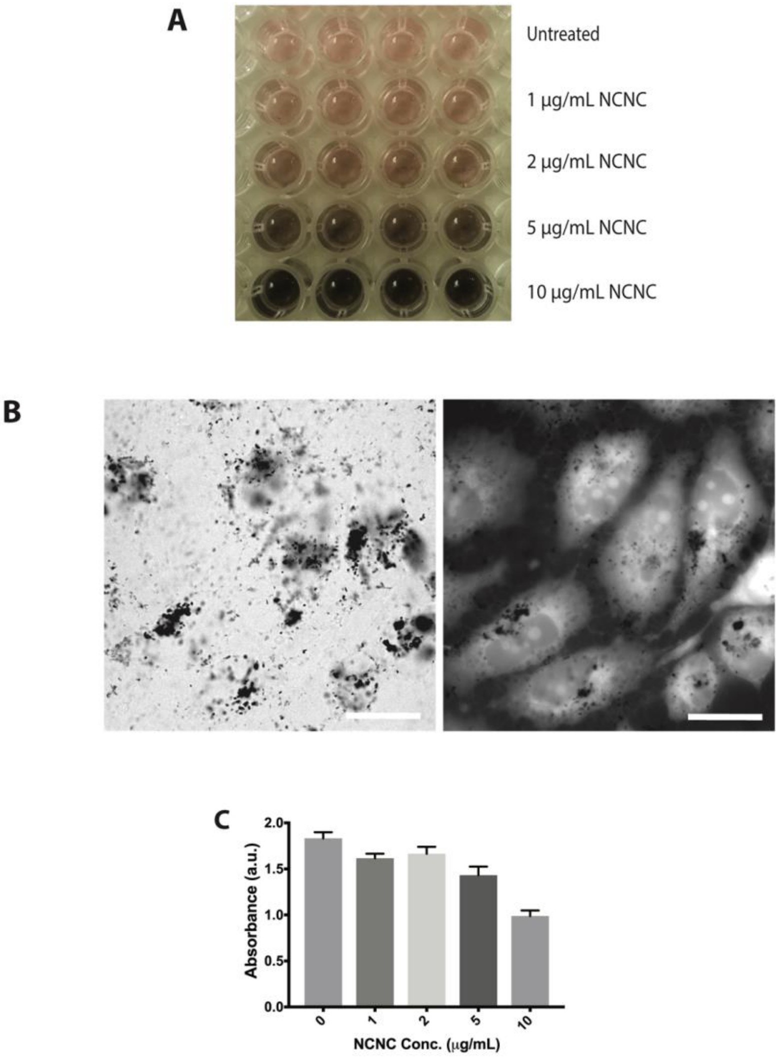

Figure S1. Cells Treatment with NCNCs. (**A**) A visual display of HeLa cells incubating in increasing concentrations of NCNCs in a 96 well plate. (**B**) HeLa cells treated with 10 µg/mL NCNC for 24 hours. Left is a bright field image of cells containing NCNCS (black spots); Right is DHE staining which aids in delineating the cell boundary. Some NCNCs can be visualized in the channel containing the DHE staining. Image was taken at 100× using an oil immersion lens. Scale bar represents 20 microns. (**C**) RPE-1 cells treated with increasing concentrations of NCNCs for 24 h. The cells were then assayed for metabolic activity using a WST-1 assay, n = 2 readings for each concentration.

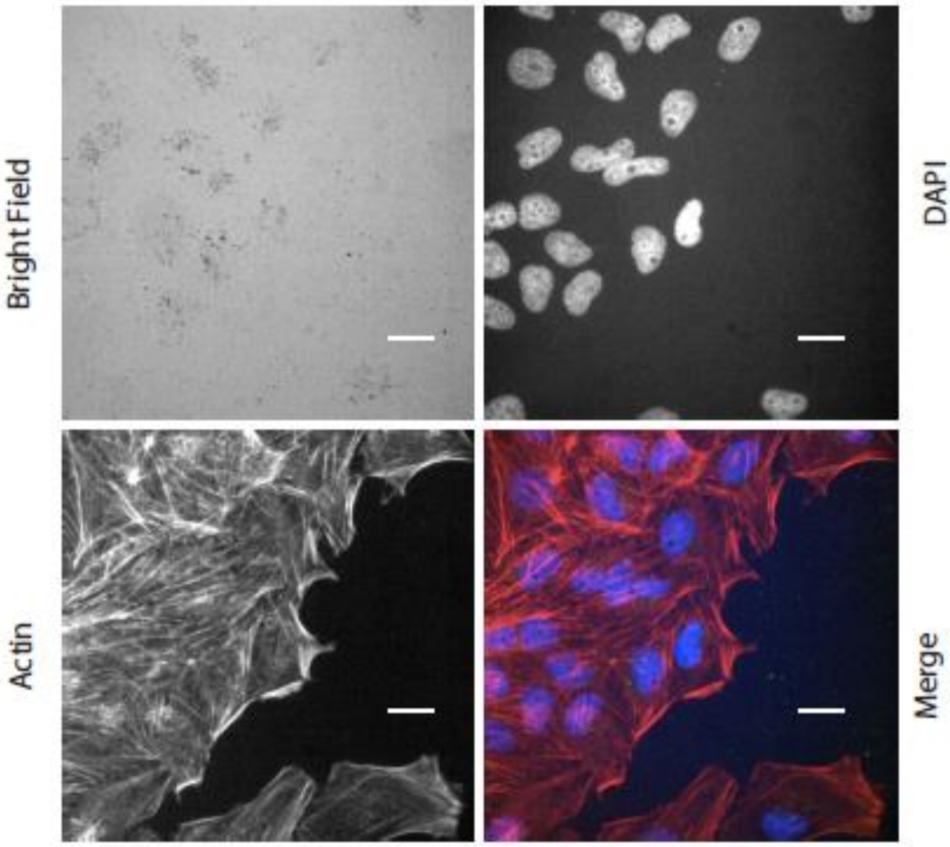

Figure S2. Fluorescence microscopy of HeLa cells treated with NCNCs. HeLa cells were treated with 2 μg/mL NCNCs for 24 h, they were then washed and stained for actin using rhodamine conjugated phalloidin. The nucleus was stained with DAPI. Scale bar indicated 20 microns.

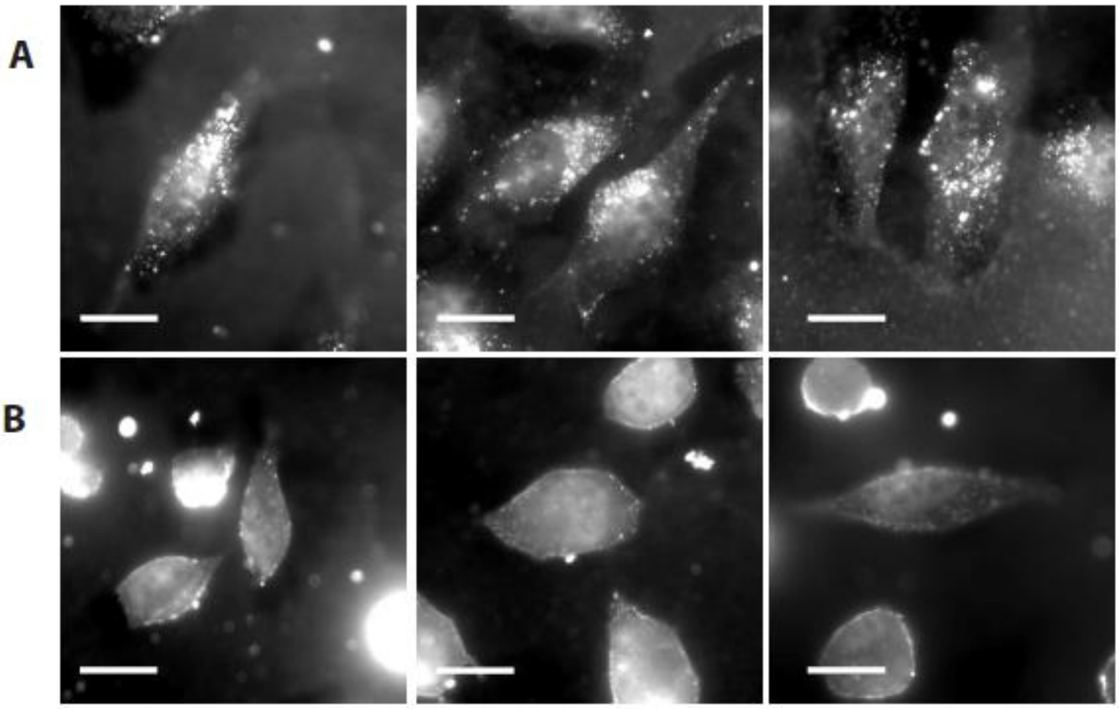

Figure S3. Dextran uptake in the presence of MβCD. (**A**) HeLa Cells incubated with Texas red dextran for 6 h. (**B**) HeLa cells were pre-incubated with 5 mM of endocytosis inhibitor MβCD for 30 min and then incubated with Texas red dextran for 6 h in the presences of the inhibitor. Scale bar indicates 20 microns.

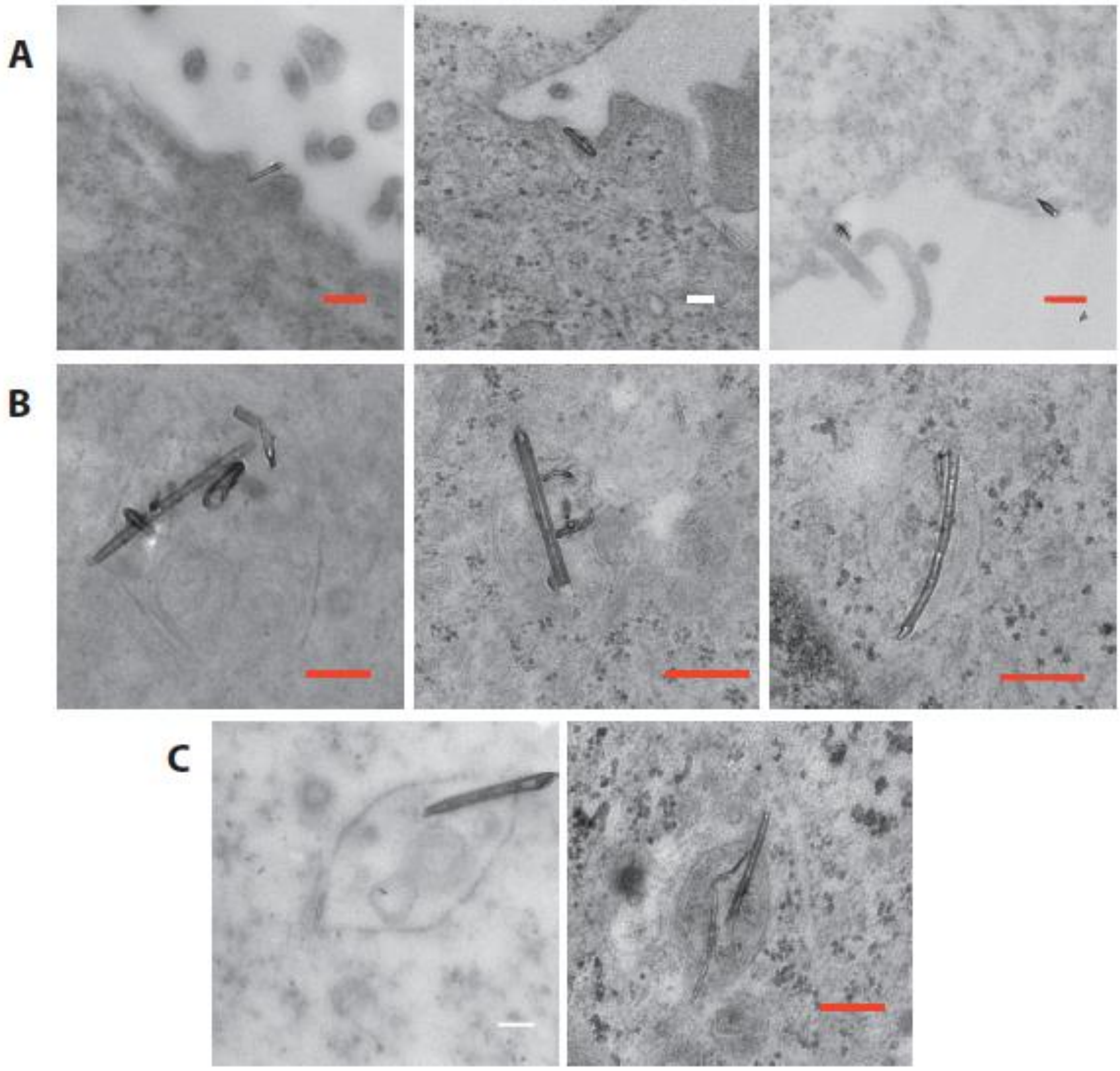

Figure S4. NCNC Passive entry and escape from vesicles. (**A**) Examples of NCNCs passing through the plasma membrane. HeLa cells were treated with 5 µg/mL for 6 h. (**B**) Examples of NCNCs distorting the membrane of vesicles. HeLa cells were treated with 5 µg/mL NCNCs for a span of 12 h (left and right) and 24 h (middle). (**C**) Examples of NCNCs passing through the membrane of vesicles. HeLa cells were treated with 5 µg/mL NCNC 12 h (left) and 48 h (right). White scale bar indicates 100 nm, red scale bar indicates 200 nm.
